# Xyloglucan Based In Situ Gel of Lidocaine HCl for the Treatment of Periodontosis

**DOI:** 10.1155/2016/3054321

**Published:** 2016-01-28

**Authors:** Ashlesha P. Pandit, Vaibhav V. Pol, Vinit S. Kulkarni

**Affiliations:** Department of Pharmaceutics, JSPM's Rajarshi Shahu College of Pharmacy & Research, Pune-Mumbai Bypass Highway, Tathawade, Pune, Maharashtra 411033, India

## Abstract

The present study was aimed at formulating thermoreversible in situ gel of local anesthetic by using xyloglucan based mucoadhesive tamarind seed polysaccharide (TSP) into periodontal pocket. Temperature-sensitive in situ gel of lidocaine hydrochloride (LH) (2% w/v) was formulated by cold method. A full 3^2^ factorial design was employed to study the effect of independent variables concentrations of Lutrol F127 and TSP to optimize in situ gel. The dependent variables evaluated were gelation temperature (*Y*_1_) and drug release (*Y*_2_). The results revealed the surface pH of 6.8, similar to the pH of saliva. Viscosity study showed the marked increase in the viscosity of gel at 37°C due to sol-gel conversion. TSP was found to act as good mucoadhesive component to retain gel at the site of application in dental pocket. Gelation of formulation occurred near to body temperature. In vitro study depicted the fast onset of drug action but lasting the release (90%) till 2 h. Formulation F7 was considered as optimized batch, containing 18% Lutrol F127 and 1% tamarind seed polysaccharide. Thus, lidocaine hydrochloride thermoreversible in situ gel offered an alternative to painful injection therapy of anesthesia during dental surgery, with fast onset of anesthetic action lasting throughout the dental procedure.

## 1. Introduction 

**Figure 1:**
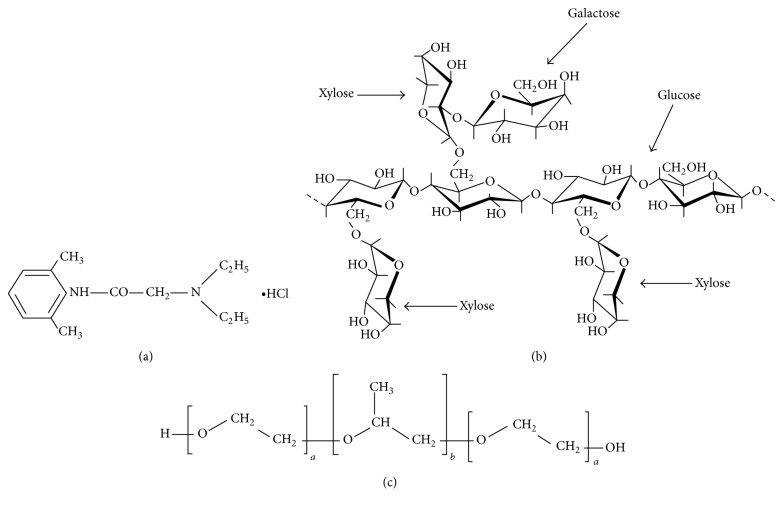
Chemical structures of (a) lidocaine hydrochloride, (b) tamarind seed polysaccharide (TSP), and (c) Lutrol F127.

Periodontosis is a serious gum infection, which affects the supporting structure of teeth such as gums, periodontal ligaments, alveolar bones, and dental cementum. Periodontosis is caused by the bacteria that stick to the surface of tooth and multiply. Toxins produced by these bacteria in plaque, formed below the gum line, irritate the gums and stimulate an inflammatory response. This results in progressive loss of alveolar bone round the teeth and thus forms the pockets (spaces between the teeth and gums) that become infected [1]. As the disease progresses, the pockets deepen and more gum tissues and bones are destroyed [2]. The number and depth of periodontal pocket vary from patient to patient. The scaling and root planning is the common practice to cure this problem. This scaling procedure needs the application of local and nerve block/infiltration anesthesia by painful needle therapy [3]. An alternative to this therapy of anesthesia is the application of gel [4]. Even though topical anesthetic gels are easy to apply, they possess some drawbacks such as tendency to spread in other areas or lower retention in plaque area, thus causing numbness of lips, tongue, and cheeks and chances of swallowing of the gel. To improve the residence time, in situ gels show promising effect [5]. In situ gel stays at application site due to increased viscosity and mucoadhesiveness and shows fast onset of action. Lutrol is a triblock polymer, consists of polyoxyethylene-polyoxypropylene-polyoxyethylene units, as shown in Figure 1(c), and forms micelles at low concentration and clear thermoreversible gel at a high concentration. The concentrated solution of Lutrol F127 (16–30%) gets transformed from low viscosity transparent solution at 5°C to a solid on heating at body temperature [6]. Formulation which consisted of Lutrol F127 forms a gel in periodontal pocket at a body temperature by modulating the gelation temperature [7]. Gel remains on application site and enhances the residence time in the periodontal pocket. It is used both internally and externally in various products that are designed for animal and human use [8]. The dental gel can be easily rinsed out with water to stop the anesthetic effect after the treatment.

Lidocaine hydrochloride (LH) (Figure 1(a)) is the first amino amide type of local anesthetics and has been in use for many years. In dentistry, it is a drug of choice to temporarily anesthetize the tiny nerve endings located on the surfaces of the oral mucosa. As a local anesthetic, lidocaine is characterized by a rapid onset of action and intermediate duration of efficacy, making it suitable for infiltration and nerve block anesthesia [9]. Lidocaine stabilizes the neuronal membrane by inhibiting the ionic fluxes required for the initiation and conduction of impulses, thereby effecting local anesthetic action.

Tamarind seed polysaccharide (TSP) is a high-molecular-weight, branched polysaccharide and consists of celluloselike backbone that carries xylose and galactoxylose substances, as shown in Figure 1(b) [10]. It is insoluble in organic solvents and dispersible in warm water to form a highly viscous mucilaginous gel with a broad pH tolerance and mucoadhesivity. In addition, it is nontoxic and nonirritant with a haemostatic activity. It is a galactoxyloglucan and possesses properties such as mucomimetic, mucoadhesive, and pseudoplastic properties [11]. TSP has been used for development of bioadhesive drug delivery systems owing to their bioadhesive properties [12]. It has been studied earlier for thermoreversible gelation property [13] and also as mucoadhesive component in mucoadhesive buccal patches for controlled release [14]. TSP is used as mucoadhesive polysaccharide polymer for systemic delivery of rizatriptan benzoate through buccal route, formulated in the form of buccal film [15]. The objective of the present study was to develop thermoreversible in situ gel of local anesthetic LH by using xyloglucan based mucoadhesive polymer TSP for insertion into periodontal pocket to have painless treatment.

## 2. Materials and Methods 

### 2.1. Materials

LH and Lutrol F127 were generously gifted by Astra Zeneca Pharma Ltd., Mumbai, India, and BASF, Mumbai, India, respectively. Tamarind seeds were purchased from local market. Triethanolamine and benzalkonium chloride were procured from Loba Chemie, Mumbai, India. All chemicals were of analytical grade and were used as received.

### 2.2. Methods

#### 2.2.1. Isolation of Mucoadhesive Agent from Tamarind Seeds

The seeds of* Tamarindus indica* were washed with water to remove the dirt and adhering material. The seeds were slightly roasted in sand and crushed to remove the outer brownish testa. Soaked seeds in water (24 h) were boiled for 1 h and kept aside for 2 h to liberate sufficient mucilage into water. Mucilage was removed from the marc by squeezing the soaked seeds through the muslin cloth. The mucilage was then isolated with equal quantity of acetone and dried at 50°C, powdered, and passed through sieve number 80 to get uniform size fine powder of TSP. The powder was stored in airtight container at room temperature till further use [16].

#### 2.2.2. Characterization of Isolated TSP

Isolated TSP was characterized for angle of repose, density, and compressibility index. Viscosity of TSP was determined to check the flow of the powder. Accurately weighed, dried, and finely powdered TSP (1 g) was suspended in 75 mL of distilled water for 5 h. Volume was made up to 100 mL to produce the concentration of 1% w/v. The mixture was homogenized by mechanical stirrer for 2 h and viscosity was determined at 500 rpm using spindle 61 and 25°C using a Brookfield viscometer (LVDVE, Brookfield Engineering Ltd., Inc., USA). The swelling index was measured to know the water holding capacity of TSP. Accurately weighed TSP (1 g) was transferred to 100 mL measuring cylinder and initial volume was noted. Distilled water was added and shaken gently. Measuring cylinder was kept aside for 24 h at room temperature. The change in volume occupied by the swelled polymer was noted. Swelling capacity of isolated polymer was expressed in terms of swelling index. Swelling index (SI) was calculated according to the following equation [17]:

(1) $$\begin{array}{c} SI=\frac{S_{1}-S_{0}}{S_{0}}\times 100, \end{array}$$

where *S*_0_ is initial volume of the powder in graduated cylindrical and *S*_1_ is the volume occupied by swollen gum after 24 h.

#### 2.2.3. Design of Experiment

The preliminary study of gel was performed by performing trial and error of batches varying the concentrations of Lutrol F127 (12 to 24%) [7, 8] and TSP (0.5 to 2.0%) [10]. The formulations revealed the effect of two factors, Lutrol F127 and TSP, on the gel formation. A 3^2^ factorial design was used to get optimized formulation of the in situ gel. Using the software Design Expert® (version 9.0), two factors were evaluated each at three levels. The concentration of Lutrol F127 (*X*_1_) and TSP (*X*_2_) was selected as independent variables (Table 1). The dependent variables evaluated were gelation temperature (*Y*_1_) and drug release (*Y*_2_).

#### 2.2.4. Formulation of In Situ Gel

Mucoadhesive in situ gel was prepared by cold method described by Schmolka [18]. In situ gel of LH (2%) was prepared using different concentrations of Lutrol F127 and TSP as shown in Table 2. Lutrol F127 and LH were dissolved in cold water by agitation. The temperature of the solution was reduced to 4°C to get the clear dispersion. Mucoadhesive TSP polymer was slowly added to the water with continuous agitation of the solution. The resulting solution was left at 4°C for 24 h to complete the polymer dissolution. Finally, benzalkonium chloride (0.001% w/v) was added as preservative. Formulation was adjusted to neutral pH with required quantity of triethanolamine. Formulations (F1 to F9) were filled in 10 mL vials, capped with rubber plugs, sealed with aluminium caps, and stored in a refrigerator (4–8°C) until further use.

#### 2.2.5. Determination of Clarity and pH

The formulations were visually checked for clarity against white and black background and categorized as follows: very clear (+++), clear (++), and turbid (+). The pH of each formulation was tested using previously calibrated pH meter (Equiptronics, EQ-610).

#### 2.2.6. Viscosity

The viscosity study of all the formulations (F1 to F9) of the in situgel was determined by the Brookfield viscometer using spindle number 61 (LVDVE, Brookfield Engineering Ltd., Inc., USA). Viscosity of the formulation was noted at two different temperatures, 25°C and 35°C, at 100 rpm shear rate.

#### 2.2.7. Gelation Temperature

Gelation temperature was measured by visual inspection method. 5 mL aliquot of gel was transferred in a test tube and placed in a water bath. With successive increments in temperature of 1°C, the samples were examined visually at which gel formed. Gel was said to have occurred, when the meniscus of the formulation would no longer move, upon tilting through right angle. Each preparation was tested thrice to control the repeatability of the measurement [8].

#### 2.2.8. Drug Content

**Figure 2:**
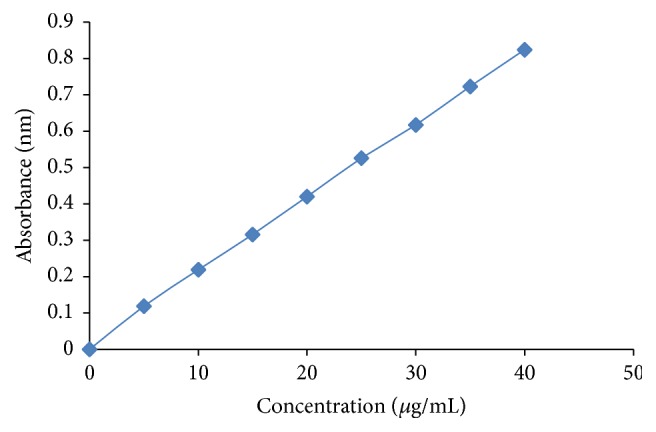
Calibration curve of lidocaine hydrochloride.

To get the drug content of gel, the formulation was maintained at 10°C, throughout the test, to remain in the liquid form. 1 mL of liquid was taken in 100 mL volumetric flask; 100 mL pH 6.8 phosphate buffer solution was added to it. Out of this, 4 mL solution was taken out into a 10 mL volumetric flask and volume was adjusted with pH 6.8 phosphate buffer. Absorbance was measured using double beam UV spectrophotometer at 263 nm (UV-1800, Shimadzu, Japan). The amount of drug present was calculated using calibration curve (Figure 2).

#### 2.2.9. Spreadability Test

Spreadability of the in situ gel was performed using a CT3 Texture Analyzer (Brookfield Engineering Lab, Inc., USA) in TPA mode. In this method an analytical probe is depressed into the sample at a defined rate to a desired depth, allowing a predefined necessary period, between the end of the first compression cycle and the beginning of second compression cycle. A cone analytical probe sample holder (TA2/1000) (30 mm diameter, 60°) was completely filled with the gel. The tapered cone was forced down into the sample holder at a defined rate of 1 mm/s and to a defined depth of 10 mm. When a trigger force of 10 g was attained, the probe proceeded to pierce the sample at a test speed of 2 mm/s to a depth of 25 mm. When the specified penetration distance was achieved, the probe departed from the sample at the posttest speed of 2 mm/s. The resulting force-time plot provided hardness, cohesiveness, and adhesiveness. The maximum force attained on the graph was a measure of the firmness of the sample at the specified depth (hardness). The maximum negative force was taken as an indication of the stickiness/cohesiveness of the sample. The work required to deform the gel in down movement of probe indicated cohesiveness. The work necessary to overcome the attractive forces between the surface of the sample and the surface of the probe provided adhesiveness of the sample [19].

#### 2.2.10. Gel Strength

**Figure 3:**
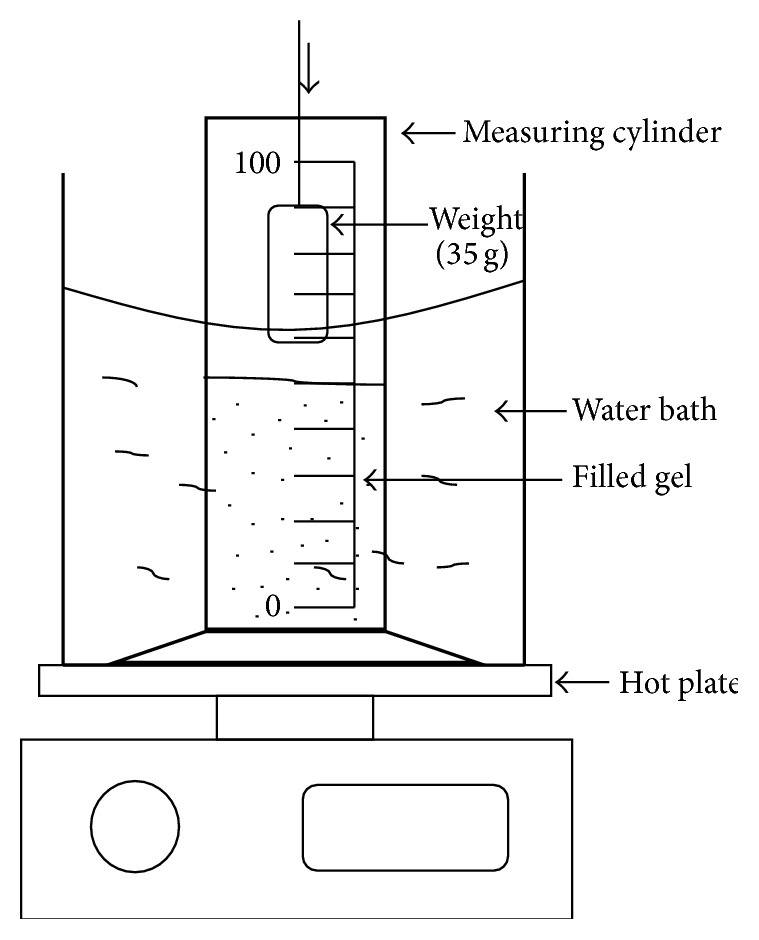
Apparatus representing the measurement of gel strength.

Gel strength is related to the viscosity of the gel. Formulation (50 g) was put in a 100 mL graduated measuring cylinder, which was further placed in thermostatically controlled water bath at 37°C. A calibrated weight of 35 g was slowly placed on the surface of the gel. Time (in seconds) required by the weight to penetrate 5 cm deep into the gel was noted [20]. The diagrammatic sketch of apparatus is as shown in Figure 3.

#### 2.2.11. Mucoadhesive Strength

Mucoadhesive strength of gel F7 was performed using a texture analyzer (CT3 Texture Analyzer, Brookfield Engineering Lab, Inc., USA). A fresh oral gum mucosal tissue of sheep was obtained from local slaughter house, cut (20 × 20 mm), and washed with phosphate buffer pH 6.8. A section of tissue was fixed on the tissue holder, keeping the orifice of the lid open to expose the small section of the tissue. Simulated saliva was placed in the 500 mL beaker and put on the thermostatically controlled heater at 37 ± 0.5°C. Tissue holder was placed in this beaker containing magnetic stirrer and equilibrated for 15 min at physiological temperature. A drop of gel was placed on the tissue through the opening of the holder. The cylinder probe (TA-5) was lowered at a rate of 0.5 mm/s until it touched the membrane. A contact force of 1 N was maintained for 60 s, and the probe was subsequently withdrawn at a rate of 0.5 mm/s to a distance of 15 mm. The maximum force required to separate the probe from the tissue being maximum detachment force in grams (*F*_max_) was noted from TexturePro CT V1.3 Build 14 software and mucoadhesive strength was determined using the following equation:

(2) $$\begin{array}{c} Mucoadhesive\;\;strength\left(\;dyne/{cm}^{2}\;\right)=\frac{F_{max}\times g}{A}, \end{array}$$

where *F*_max_ is the maximum detachment force in grams, *g* is acceleration due to gravity, and *A* is the area of tissue exposed to the gel.

#### 2.2.12. In Vitro Release Studies

In vitro release study of formulations (F1 to F9) was performed using dialysis membrane (Himedia, India). Dialysis membrane consisted of cellophane membrane having an average flat width of 24.26 mm, average diameter of 14.3 mm, and capacity of approximately 1.61 mL/cm, utilized for diffusion. Prior to the diffusion study, the dialysis membrane was soaked overnight in pH 6.8 phosphate buffer solution. Formulation (1 mL) was placed in the dialysis membrane, cut off in the size of 7 cm length, and sealed on both sides. The dialysis tube was then placed in a glass beaker containing 20 mL of pH 6.8 phosphate buffer solution equilibrated at 37 ± 0.5°C [12]. 1 mL of aliquot was withdrawn after every 10 min till 2 h to get the amount of drug released through the membrane and entered in the phosphate buffer and replaced with same volume of preheated solution to maintain the sink condition. After suitable dilutions, samples were analyzed UV spectrophotometrically at 263 nm.

#### 2.2.13. Ex Vivo Study

The gel permeated through the oral tissue was performed using ex vivo study. A fresh gum mucosal tissue was carefully removed from the oral cavity of the sheep, obtained from the local slaughter house. The mucosa was stored in the saline solution. The mucosa was cut off in circular shape of 3.5 cm in diameter and fixed in between the donor and the receptor compartment of the Franz diffusion cell, keeping mucosal side up. Prior to study, the mucosa was equilibrated by putting in phosphate buffer pH 6.8 for 1 h. The optimized batch gel formulation (equivalent to 10 mg of LH) was applied evenly on the mucosal membrane. The receptor compartment was filled with 25 mL of pH 6.8 phosphate buffer solution maintained at temperature 37°C. The assembly was put on the magnetic stirrer. At predetermined time periods of 30 min time interval, 1 mL of aliquot was withdrawn from the receptor compartment, replacing the same volume with pH 6.8 phosphate buffer of temperature 37°C, for a period of 2 h. After suitable dilution, samples were analyzed UV spectrophotometrically at 263 nm.

#### 2.2.14. Stability Study

The optimized batch was packed in amber colour bottle, sealed, kept at 40°C, and maintained at 75% relative humidity in stability chamber (Thermolab, India) for a period of 3 months. Samples withdrawn at 1, 2, and 3 months were characterized for appearance, drug content, and in vitro drug release.

## 3. Results and Discussion

### 3.1. Characterization of TSP

The percentage yield of isolated tamarind seed polysaccharide was 70.3% w/w. The powder obtained was cream in colour with good swelling index (200%) and viscosity (aqueous dispersion of 1% w/v was 8.85 cps). Angle of repose, bulk density, tapped density, and Carr's index revealed good flow properties.

### 3.2. Design of Experiment

Experimental trials were performed for nine possible formulations suggested by 3^2^ factorial design. Mathematical treatment of the nine possible combinations of batches F1–F9 is shown in Table 2.

### 3.3. Clarity, pH, Drug Content, and Viscosity

All formulations (F1 to F9) showed the clear visibility of the gel. The pH of all formulations was found to be in the range of 6.5–6.8, which was similar to the normal pH of mouth saliva (6.8 to 7.4) [2]. Scanning of lidocaine HCl solution in pH 6.8 phosphate buffer solution by UV spectrophotometer showed *λ*_max_ at 263 nm. At this wavelength the standard curve followed Beer-Lambert's law in the concentration range of 5 to 30 *µ*g/mL with *R* ^2^ = 0.9992. The drug content of all formulations was found to be in the range of 93–99%, thus confirming the uniformity in formulation of the gel (Table 3). As the concentration of the Lutrol F127 (18–22%) and TSP (0.5–1.5) was increased, the viscosity of the gel was also increased. Formulations with the higher concentration of TSP (0.5 to 1.5%) at the same amount of Lutrol F127 (22%) in F8, F5, and F4 showed the increase in viscosity at both temperatures (25°C and 35°C). Formulations consisted of the increased amount of Lutrol F127 (14 to 22%) at the same amount of TSP (1.5%) in F3, F2, and F4 and showed the increase in viscosity (Table 3). Thus, the viscosity of formulations in gel state was found to be proportionate with the increase in concentration of Lutrol F127 and TSP.

### 3.4. Gelation Temperature

Gelation temperature range suitable for dental gel is 33–35°C (Table 3), which means a gel should be in liquid form at room temperature and form a gel phase in the buccal cavity. If the gelation temperature of liquid gel is lower than 33°C, gelation occurs at room temperature, leading to difficulty in administering the formulation. If the gelation temperature is higher than 35°C, the gel remains in a liquid form at physiological temperature, resulting in leakage from the periodontal pocket. The gelation temperature of formulations F1 to F9, inspected visually, was found to be within the range of 20 to 37°C. The data presented in Table 3 clearly indicated that the gelation temperature was strongly dependent on the concentration of selected independent variables. An increase in concentration of Lutrol F127 and TSP decreased the gelation temperature. This effect of variables was further supported by the data of design of experiment.

#### 3.4.1. Effect of Formulation Variables on Gelation Temperature

A mathematical relationship between factors and levels was studied by response surface regression analysis using Design Expert (version 9.0.3.1) software. Equation (3) shows the relationship between the variables and response of gelation temperature (*Y*_1_).

Equation (3), in the form of coded values, is as follows:

(3) Y1=+35.14−8.17X1−0.83X2−0.50X1X2−5.79X12+0.21X22,

where *Y*_1_ is the gelation temperature, *X*_1_ is concentration of Lutrol F127, and *X*_2_ is concentration of TSP.

A negative sign before a factor in polynomial equation (3) indicated that the response has reciprocal effect on both factors. Influence of factors *X*_1_ and *X*_2_ on *Y*_1_ was best fitted to quadratic model and found to be significant with *F* value of 345.01 (*p* < 0.05). Variables *X*_1_ and *X*_2_ have *p* value of 0.000404 (*p* < 0.05) and 0.0061. The variables, which have *p* value less than 0.05, significantly affect the gelation temperature. The predicted *R* squared value of 0.9828 was in reasonable agreement with the adjusted *R* squared value of 0.9962. Analysis of variance (ANOVA) was applied to determine the significance and the magnitude of the effects of the variables and their interactions.

As the temperature of Lutrol F127 was increased, the copolymer molecules aggregate to form micelle. This micellization occurs due to the dehydration of hydrophobic propylene oxide blocks. This represents the very first step in the gelling process. The gel formation occurs when the concentration is above the micellar concentration (50%) [21]. This gelation was attributed to the ordered packing of micelles. Interestingly, addition of mucoadhesive polymer (TSP) lowered the gelation temperature of the gel. The viscous nature of TSP may be responsible for lowering the temperature. A micellar association for Lutrol F127 occurs over the temperature range of 10–40°C. Critical micellar temperature is nearer to physiological temperature [7]. These results are in close agreement with the data obtained for in situ gel formulated for periodontal disease using Carbopol 934 P and poloxamer 407 [22].

**Figure 4:**
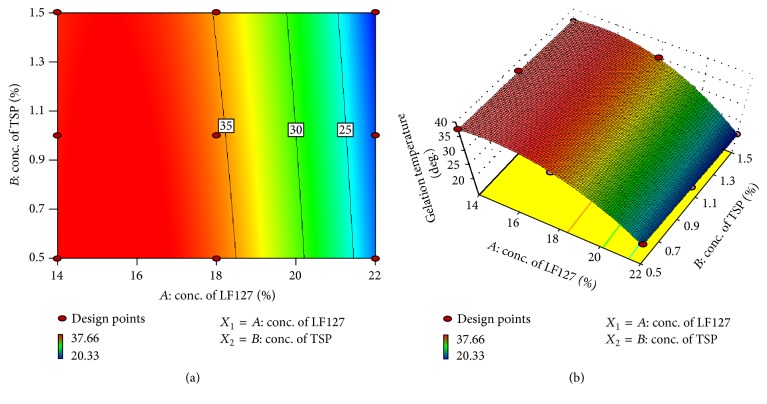
Counter plot (a) and three-dimensional response surface plot (b) showing the effect of factors on gelation temperature.

The regression model obtained was used to generate the counter plots for analyzing interactions of the independent factors. Counter plot shown in Figure 4(a) suggested the correlation between the two variables. Gelation temperature of gel decreased with increase in concentration of Lutrol F127 but showed much less effect on change in concentration of TSP, which was indicated clearly in vertical axis of counter plot. The combined effect of factors *X*_1_ and *X*_2_ can be further elucidated with the help of three-dimensional response surface plot as shown in Figure 4(b). High level of factor *X*_1_ showed reduction in gelation temperature and low level showed higher gelation temperature, which indicated that factor *X*_1_ has significant negative effect on gelation temperature of gel. Factor *X*_2_ (TSP) was found to have much less reciprocal effect on gelation temperature. Increase in concentration of TSP lowered the gelation temperature of in situ gel.

### 3.5. In Vitro Drug Release

**Figure 5:**
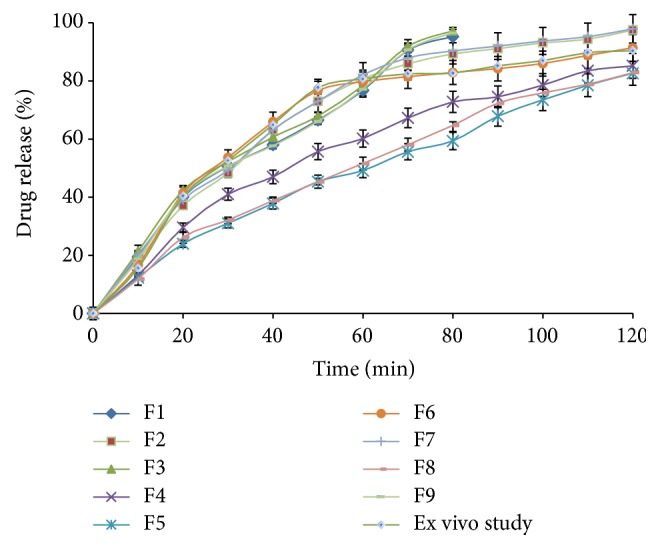
Drug release study of in situ gel.

Formulations F1 to F9, subjected to in vitro release study, are represented graphically in Figure 5. Formulations F1, F3, and F9 showed 95% of drug release within 80 min. It was observed that, at less concentration of Lutrol F127 (14%) and higher amount of TSP, the initial rate of drug release was very rapid due to incomplete gel formation. Further, increase in concentration of Lutrol F127 to 18% (F2, F6, and F7) delayed the drug release and released more than 95% of the drug within 2 h. However, further more amount of Lutrol F127 (22%) in F4, F5, and F8, the drug release was retarded and was not released (82%–85%) completely even after 2 h, which is maximal time needed for dental surgery to have anesthetic action.

Presence of TSP in in situ gel was found to affect the drug release. As the amount of TSP was increased from 0.5% (F9, F6, and F8) to 1.0% (F1, F7, and F5) and 1.5% (F3, F2, and F4), drug release was found to be increased. Hence it was concluded that the concentration of Lutrol F127 should not exceed 18% [7, 22]. TSP should be at optimal concentration of 1.0–1.5%.

#### 3.5.1. Effect of Formulation Variables on Drug Release

Analysis of variance was applied to determine the significance and the magnitude of the effects of the variables and their interactions on drug release. The obtained regression model was used to generate the counter plot for analyzing the interactions of the independent variables on dependent variable. Equation (4) shows the polynomial equation in terms of coded levels obtained for drug release:

(4) $$\begin{array}{c} Y_{2}=+91.80-6.47X_{1}+1.67X_{2}. \end{array}$$

**Figure 6:**
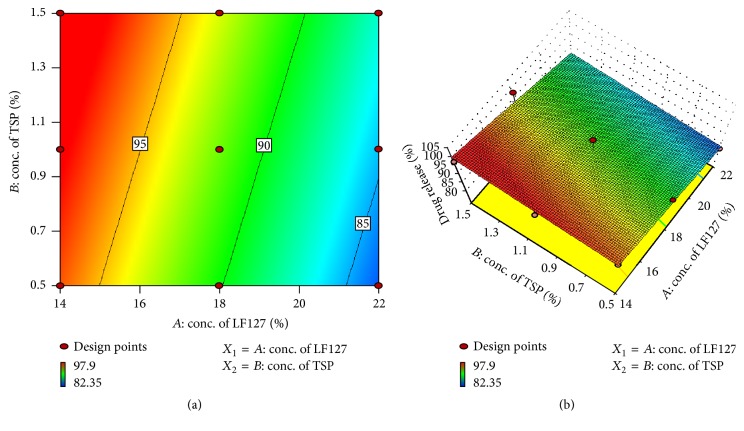
Counter plot (a) and three-dimensional response surface plot (b) showing the effect of factors on drug release.

The contour plot and three-dimensional analysis showed that drug release was decreased with increase in concentration of Lutrol F127 and increased with increase in concentration of TSP.  Figure 6 indicates scale from blue colour to red colour. When colour of response surface shifts from blue towards red it indicates increase in percentage of drug release and vice versa. The same effect was reflected in (4) showing negative sign before *X*_1_ and positive sign before *X*_2_. The variable *X*_1_ showed higher numerical value than *X*_2_, which confirmed the more negative effect of Lutrol F127 compared to the positive effect of TSP on drug release of in situ gel. ANOVA results confirmed the adequacy of the linear model for drug release (*Y*_2_) and were found to be significant with *F* value of 9.19 (*p* < 0.05). The predicted *R* squared value of 0.5491 was in reasonable agreement with the adjusted *R* squared value of 0.6719.

### 3.6. Gel Strength

Formulations (F1 to F9) showed good gel strength in the range of 40 to 55 s. Higher gel strength which was related to viscous polymer verified the retaining capacity of gel in the periodontal pocket. F4, F5, and F8 needed more time (55 s, 52 s, and 48 s, resp.) to penetrate the weight in it, compared to the other formulations.

### 3.7. Spreadability Testing

**Figure 7:**
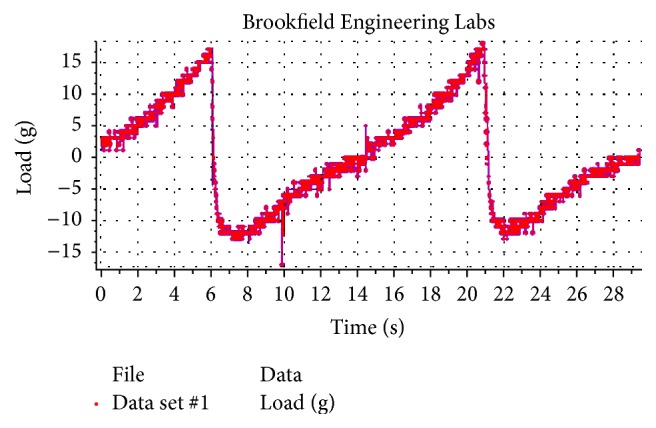
Graph of spreadability of gel on texture profile analysis.

Spreadability denotes the extent of area to which the gel readily spreads on its application. Hence, it is a property related to the viscosity of mucoadhesive polymer. The greater the viscosity is, the lesser the spreadability is [8] and the more the retention of gel in the dental pocket can be. Texture profile analysis spectra of in situ gel of formulation (F7) showed the hardness of 16.3 g, cohesiveness of 0.81, and adhesiveness of 0.4 mJ (Figure 7). These results expressed the applicability of gels to site of application or adhesivity and indicated the retention time of the gel on the site of application. The more negative value indicated higher adhesivity of the sample. Hardness value confirmed the good firmness of gel and cohesiveness value indicated the better consistency of the gel. As the probe returned to its starting position, the initial lifting of the weight of the sample on the upper surface of the disc produced the negative part of the graph. This indicated the cohesiveness and resistance of the sample to be separated (flow off) from the disc. The maximum negative force on the graph indicated the sample adhesive force.

### 3.8. Mucoadhesive Strength

Gel formulation (F7) showed good mucoadhesive strength (1,124 dyne/cm^2^) to the sheep mucosa, needed to hold the gel in the dental pocket during surgery, to show therapeutic anesthetic action.

### 3.9. Ex Vivo Study

Drug permeation through the oral tissue was performed though the sheep oral mucosa using Franz diffusion cell. The liquid gel was immediately converted to solid gel after putting on the oral mucosa, maintained at 37°C. Initially, faster drug release was observed which was due to the incomplete gel formation. This faster release was actually found to be good to attain faster anesthetic action at the start of dental procedure. As the time progressed, the gelation temperature (35.33°C) was achieved and release rate was slowed down. The gel was retained on the mucosa, which confirmed good mucoadhesion using TSP polymer. The formulation exhibited the good release of LH (98.05%), as shown in Figure 5. The release was found to be in good correlation with in vitro study (97.5%).

### 3.10. Stability Study

Accelerated stability study of an optimized batch of in situ gel (F7) was carried out as per ICH guidelines. There was no significant change in drug content, pH, viscosity, gelation temperature, and drug diffusion for the selected formulation, F7, after 90 days at 40°C ± 0.5/75%  ± 5% RH. The drug (92.7%) was diffused through the dialysis membrane within 2 h.

### 3.11. Optimization of Formulation

The computer optimization technique by the desirability approach was used to produce the optimum formulation. The process was optimized for the response variables *Y*_1_ and *Y*_2_. The optimized formula was reached by setting maximum percentage of drug release at 2 h and optimal gelation temperature. Formulation F7 was found to be optimized formulation which contained 18% Lutrol F127 and 1% TSP.

## 4. Conclusion

Lidocaine hydrochloride loaded periodontal temperature-sensitive in situ gel was successfully developed by cold method using xyloglucan based mucoadhesive polymer TSP for insertion into periodontal pocket to have painless treatment. Viscosity study showed the marked increase in the viscosity of gel at 37°C due to sol-gel conversion. Gelation of formulation was observed near to body temperature. In vitro study depicted the rapid onset of drug action, extending till 2 h, to cover period of periodontal treatment. Use of natural, less costly, biodegradable, and easily available mucoadhesive TSP polymer as well as avoidance of needle insertions during scaling and root planning of periodontosis helped achieve patient compliance by ultimately reducing the cost of the treatment. TSP (1%) and Lutrol F127 (18%), in combination, imparted viscous behaviour to gel needed to retain the formulation in periodontal pocket. Thus, lidocaine hydrochloride thermoreversible in situ gel offered an alternative to painful injection therapy of anesthesia during dental surgery, with rapid onset of anesthetic action lasting throughout the dental procedure.

## Figures and Tables

**Table 1:** Coded levels of factorial design.

Factor	Level		
	(−1)	(0)	(+1)
Concentration of Lutrol F127 (%) (*X*_1_)	14	18	22
Concentration of TSP (%) (*X*_2_)	0.5	1.0	1.5

**Table 2:** Composition of lidocaine HCl gel formulations.

Ingredient	F1	F2	F3	F4	F5	F6	F7	F8	F9
Lidocaine (%)	2	2	2	2	2	2	2	2	2
Lutrol F127 (%)	14	18	14	22	22	18	18	22	14
TSP (%)	1.0	1.5	1.5	1.5	1.0	0.5	1.0	0.5	0.5
Triethanolamine (mg)	q.s.	q.s.	q.s.	q.s.	q.s.	q.s.	q.s.	q.s.	q.s.
Benzalkonium chloride (%)	0.001	0.001	0.001	0.001	0.001	0.001	0.001	0.001	0.001
Purified water (mL)	10	10	10	10	10	10	10	10	10

**Table 3:** Formulation table.

Formulation code	Clarity	pH	Viscosity (cps) (±SD)		Drug content (%) (±SD)	Gelation temperature (°C) (±SD)
			At 25°C	At 35°C		
F1	Transparent	6.6	233.66 ± 0.57	445.66 ± 6.02	96.5 ± 0.8	37.66 ± 0.3
F2	Transparent	6.8	1422.66 ± 3.5	1634.67 ± 4.16	97.10	35.33
F3	Transparent	6.7	245.0 ± 1	315.33 ± 3.05	95.20	36.66
F4	Transparent	6.6	2760.33 ± 2.51	2957.0 ± 5.67	93.30	20.33
F5	Transparent	6.5	2438.0 ± 8.54	2781.66 ± 4.04	94.60	21.33
F6	Transparent	6.8	934.66 ± 2.08	1322.33 ± 2.51	97.80	35.66
F7	Transparent	6.8	1211.0 ± 10.14	1636.0 ± 5.29	98.10	35.33
F8	Transparent	6.6	2057.33 ± 5.03	2619.33 ± 6.11	94.95	22.66
F9	Transparent	6.7	249.66 ± 222.51	363.67 ± 3.51	96.20	37.66

±SD: Standard Deviation.
